# Bringing Big Data to Bear in Environmental Public Health: Challenges and Recommendations

**DOI:** 10.3389/frai.2020.00031

**Published:** 2020-05-15

**Authors:** Saskia Comess, Alexia Akbay, Melpomene Vasiliou, Ronald N. Hines, Lucas Joppa, Vasilis Vasiliou, Nicole Kleinstreuer

**Affiliations:** ^1^Department of Environmental Health Sciences, Yale School of Public Health, New Haven, CT, United States; ^2^Department of Statistics and Data Science, Yale University, New Haven, CT, United States; ^3^Symbrosia Inc, Kailua-Kona, HI, United States; ^4^US Environmental Protection Agency, Center for Public Health and Environmental Assessment, Research Triangle Park, NC, United States; ^5^Microsoft Corporation, AI for Earth, Redmond, WA, United States; ^6^National Toxicology Program Interagency Center for the Evaluation of Alternative Toxicological Methods, National Institute of Environmental Health Sciences, Research Triangle Park, NC, United States

**Keywords:** artificial intelligence, public health, machine learning, open data, environmental health sciences, big data

## Abstract

Understanding the role that the environment plays in influencing public health often involves collecting and studying large, complex data sets. There have been a number of private and public efforts to gather sufficient information and confront significant unknowns in the field of environmental public health, yet there is a persistent and largely unmet need for findable, accessible, interoperable, and reusable (FAIR) data. Even when data are readily available, the ability to create, analyze, and draw conclusions from these data using emerging computational tools, such as augmented and artificial intelligence (AI) and machine learning, requires technical skills not currently implemented on a programmatic level across research hubs and academic institutions. We argue that collaborative efforts in data curation and storage, scientific computing, and training are of paramount importance to empower researchers within environmental sciences and the broader public health community to apply AI approaches and fully realize their potential. Leaders in the field were asked to prioritize challenges in incorporating big data in environmental public health research: inconsistent implementation of FAIR principles in data collection and sharing, a lack of skilled data scientists and appropriate cyber-infrastructures, and limited understanding of possibilities and communication of benefits were among those identified. These issues are discussed, and actionable recommendations are provided.

## Introduction

Out of the tens of thousands of individual chemicals currently in commerce (and many more mixtures, natural products, and metabolites) <10% have been screened for safety. The U.S. EPA's Toxic Substances Control Act (TSCA) Chemical Substances Control Inventory contains roughly 85,000 chemicals (U.S. EPA, 2016), and the European Chemicals Agency (ECHA) Inventory lists over 100,000 unique substances (as of the most recent update in August 2017), of which ~22,000 are registered substances with some information on structure, usage, or toxicity (ECHA, 2017). Understanding which chemicals in the environment, both with and without safety data, pose a risk to human health requires that we more effectively leverage the data that we already have, and that we take intelligent approaches to generating new data. While the traditional means of collecting chemical safety data (animal models) are laborious and of variable accuracy and human relevance (Hartung, 2009), such reference data can still be used to train models for prioritizing and predicting toxicity of new chemicals, provided the data are curated in a computationally accessible format and, ideally, integrated with other lines of evidence providing mechanistic information. This requires significant effort, both in collecting, and extracting information as well as annotating it appropriately.

These toxicological problems are mirrored in public and environmental health more generally: huge, complex issues with inadequately curated data, and analytic power. Recent research in toxicology has focused on high-throughput screening to rapidly produce quantitative data on thousands of human biological targets (e.g., Thomas et al., 2019), data-mining to identify relevant end-points building predictive models for adverse toxicological outcomes (e.g., Saili et al., 2019), and application of cutting-edge machine learning (ML) and artificial or augmented intelligence (AI) techniques (e.g., Luechtefeld et al., 2018). Collectively, these technologies facilitate enhanced mechanistic insights and may obviate the need for inefficient testing in animal models, but they are still not considered mainstream approaches nor are they widely accepted by regulatory agencies. Individual research programs generate large data sets, but without centralized coordination, standardized reporting, and common storage structures, the data cannot be effectively combined and used to its full potential. The federal Tox21 research consortium has, to date, tested more than 9,000 chemicals to varying degrees in 1,600 assays and demonstrated environmental chemical interactions with critical human and ecologically-relevant targets (Tice et al., 2013). Translational systems approaches are being employed by this and other programs (e.g., Horizon 2020, EUToxRisk, CEFIC LRI, OpenTox) to produce diverse data streams and predict chemical effects on human health and disease outcomes (e.g., Kleinstreuer et al., 2014). At the same time, there have been substantial efforts to develop and deploy sensors and satellite systems that yield additional large and complex data sets that provide information about chemical exposures (Dons et al., 2017; Ring et al., 2019; Weichenthal et al., 2019). Further, epidemiologists are actively developing ML and AI approaches to enhance understanding of chemical exposures and associated disease risks (Brandon et al., 2018). However, these efforts also are largely disconnected from one another and operate independently, despite the clear potential benefits if such data could be combined and jointly analyzed. Given the need to integrate and analyze large, multifactorial data sets, researchers in public health and the environmental health sciences (EHS) would greatly benefit from the ability to collect, process, analyze, and make inferences on data using ML and AI. However, in these fields, a general lack of relevant knowledge among many researchers, sparse, distributed, or inaccessible data, and an inadequate framework for sharing and disseminating innovations impede efforts to implement these approaches. Here, we discuss three specific areas with room for improvement in the public health/EHS field: data collection and sharing, researcher knowledgebase, and a recognition of the benefits AI/ML can bring to current problems. Recommendations are provided in each of these areas to facilitate bringing big data to bear on public health and EHS challenges.

## Data Collection and Sharing

### Challenge

A major hurdle confronting investigators conducting public health and EHS research is a lack of comprehensive human and environmental exposure and effects data that are annotated using controlled vocabularies. Addressing this problem is a prerequisite to applying AI and ML, as without sufficient, high-quality data and metadata, the analytic methods themselves are irrelevant. Quantifying environmental exposure, such as from air, water, soil, and food, is difficult both at the micro (localized to individuals and small geographic units) and macro (national and international) levels. For instance, air pollution can vary up to eight-fold within a given city block, but most U.S. cities have only one air quality monitor (EDF, 2019). Epidemiologic studies of air pollution health effects often must rely on disparate data that lack both temporal and spatial specificity and cannot account for the movement of people across different areas of pollution. Without continuous and advanced monitoring, and robust computer modeling methods, illnesses related to transient exposures might not be recognized as part of a significant pattern until substantial adverse health effects have occurred. This is one example where the development of AI tools in the EHS space has been hindered not by the AI technology capacity itself but instead by a lack of reliable, interconnected data (NAS, 2019). This is equally true in the medical sector with respect to patient treatment and outcomes. IBM's ambitious partnership with the MD Anderson Cancer Center to develop AI to expedite clinical decision-making has been at a standstill after years of development due to a lack of standardized, accessible data (Jaklevic, 2017).

Even when standardized data are available, finding, accessing, and processing it can be a monumental task. The absence of a uniform framework for openly sharing and storing data means that researchers devote significant time to locating relevant data. Knowledge of where to find data is often highly sector-specific, inhibiting cross-disciplinary research. For example, a climate scientist interested in public health would need knowledge of health-specific data repositories to conduct the search. Rather than waste manual effort and time in locating data, let alone integrating it, coordinated efforts could result in processes that could be automated and simplified. Ethical concerns have been voiced in regard to organizing large repositories of these types (Ienca et al., 2018). Of these, patient data privacy is a major concern, and breaches of patient records databases are a constant challenge. Unique patient identifier numbers and other de-identification/anonymization techniques can protect patient privacy, while allowing for meaningful research and analysis (Emam et al., 2015). New encryption based techniques allow for predictive modeling while maintaining the privacy of sensitive information, such as the application of homomorphic encryption to patient data in predicting cardiovascular disease (Bos et al., 2014). However, inconsistent regulations and lack of practical protocols around handling sensitive information have resulted in unethical scenarios, where data is being sourced from countries where patients have minimal rights (Mittelstadt and Floridi, 2016). Not only is this problematic from an ethical perspective, it also limits AI innovation to only those who have access to these obscure datasets. Specific tools developed by startups who have the luxury of sourcing data from elsewhere are often acquired by large corporations, making innovation an exclusive pursuit. Thus, the environmental public health field requires a revolution in the collection and organization of environmental exposure and effects data as a first step in democratizing information access and building better models to improve predictions.

### Recommendations

Further work is clearly needed in data collection and sharing, but recent attempts in specific sectors are exemplar in the aggregation of data and development of open, accessible repositories that maintain necessary privacy standards. In 2016, over 50 contributing researchers from global institutions proposed the “Findable, Accessible, Interoperable, and Reusable” (FAIR) Guiding Principles for scientific data management and stewardship (Wilkinson et al., 2016). These principles bridge the divide between human-conducted and machine-driven research behaviors. Using FAIR principles, the NIH is creating Data Commons, a platform for data management, and metadata cataloging for terminologies and ontologies (Mahony et al., 2018). This framework has been one of the key drivers behind new repositories and tools such as the National Toxicology Program's Integrated Chemical Environment (ICE) (Bell et al., 2017) portal and the U.S. EPA's CompTox Chemicals Dashboard (Williams et al., 2017), which allow FAIR principles to be applied to non-animal *in vitro* and *in silico* data, along with *in vivo* animal data and human exposure information. A collaboration between the US FDA, the non-profit Clinical Data Interchange Standards Consortium (CDISC), and other stakeholders, resulted in the development of study data standards for non-clinical, clinical, analysis and metadata (https://www.cdisc.org/standards) to create common reporting formats. These concepts are cornerstones of the 2018 U.S. Strategic Roadmap for Modernizing Safety Testing of Chemicals and Medical Products, developed by 16 U.S. federal agencies, which advocates for practices that increase confidence in new data-driven research methods (ICCVAM, 2018). A significant portion of the work done by these data powerhouses is retrospective data curation, often performed manually (e.g., Kleinstreuer et al., 2016). Work is ongoing to automate some aspects of the information extraction pipeline, but additional efforts to standardize reporting formats, and metadata terminologies in emerging research could lighten the curation burden on these institutions and streamline data annotation and storage, allocating greater resources to the development of novel applications.

Many of the recent advances in developing openly accessible databases of environmental exposure information have come from the private sector, often in partnership with non-profit organizations and academic institutions. The Monarch Initiative (https://monarchinitiative.org/) is one such collaboration to apply ontologies, or semantic descriptions, to disease phenotypes and enable intra- and inter-species comparisons and connection to genotypes, pathways, and experimental models. Another example is a pilot project in Oakland, California, between the Environmental Defense Fund (EDF) and Google Earth Outreach which involved attaching air quality sensors to Google Street View cars. This was recently extended to a partnership with an environmental sensor company (Aclima) to equip Street View cars with mobile air quality sensors in cities around the world (Business Wire, 2018). The sensors capture detailed air quality and emissions data at high (street-block level) resolution and temporal frequency. These data will be aggregated and made available on a Google database. Google also recently announced that it will report estimates of city-level greenhouse gas emissions and annual driving, biking, and transit ridership (data gathered via Google Maps and Waze) (Meyer, 2018; Google, 2019). Google's new Dataset Search is an initial attempt to apply distributed search to datasets from the environmental and social sciences, government data, and news organizations (Noy, 2018). By providing access to data from multiple disciplines via a single platform, researchers can conduct interdisciplinary work fundamental to environmental and public health (Vincent, 2018). Applying these powerful methods to better curate and integrate diverse sources of data will promote greater understanding of complex and dynamic systems. However, acceptance and implementation of these improvements are not yet widespread, particularly in the public and environmental health sectors. Following the lead of these innovative pilot studies, a greater emphasis needs to be placed on developing the appropriate infrastructure for effective, standardized data collection approaches, common ontologies, and uniform sharing protocols.

## Researcher Knowledge Base

### Challenge

Public health and EHS researchers are not traditionally trained in scientific computing and data science, and computer scientists do not typically apply their skill sets to EHS problems. This impedes the introduction and utilization of powerful data science techniques to public and environmental health practice and research. The American Association for Public Health, the body responsible for accrediting public health programs in the United States, currently does not include computer skills in the core competencies required of a Master's of Public Health (MPH) program. This omission is a disservice both to public health students and the field of public health research more broadly. Just as being able to read and write are fundamental skills and required baseline competencies for entering a graduate program, computer science will eventually become a similar prerequisite for comprehensive and effective analytical approaches across the biological and toxicological realm. Ultimately, the EHS and public health disciplines need a culture and skill shift. Over the next decade, scientists will need to understand the fundamentals of computer and data science in order to be successful in their field. Having this core computer science competency will be critical to the future success of transdisciplinary research in the EHS and public health disciplines.

### Recommendations

Current public health and EHS students are interested in these issues, which means that public health schools need to integrate computer and data science into their core curriculums. While students interested in big data and public health are not at a total loss for resources, provided they are willing to seek them out, there are major gaps in the academic arena. There are a handful of existing programs that currently provide the skill set needed to apply data science to public health research. The Master of Science in Data Science is an option offered from a number of Universities, but few offer, or even consider, the integration of this degree with applications in the field of public health. Within the status quo, students are presented with the option of either an MPH or an MS in Data Science, with little crossover between the two. Even programs such as Harvard's Health Data Science MS, which is offered through the School of Public Health, only requires one epidemiology course and then places the onus on the student to integrate the public health perspective into their own research.

The lack of computer skills in the core competencies of the MPH is one example of an area with obvious and immediate room for improvement. However, the need for cross-disciplinary training and communication is equally relevant from the perspective of computer scientists, who should be educated and engaged in EHS and public health applications. Students from both academic disciplines should be encouraged, if not required, to engage in coursework in the other. This idea of “cross-departmental partnerships” would arm public health students with the technical skills needed to integrate computer science, AI, and ML into their work while providing insight for potential EHS projects to which computer science students could apply their skills. The thoughtful design of such a program also would foster a culture of transdisciplinary research which will be key to finding solutions to complex environmental and public health problems. The Massachusetts Institute of Technology is laying the necessary groundwork for such a program by creating a new college for AI. With a $1 billion investment, the college is expected to start in the Fall of 2019 with the goal of integrating AI systems across academic fields (Lohr, 2018). Continuing education programs, such as the New Approach Methodology Use in Regulatory Application training series (https://www.pcrm.org/ethical-science/animal-testing-and-alternatives/nura), are being offered to ensure that environmental public health professionals also begin to develop the skills and expertise needed to leverage big data and implement AI and ML based approaches. A highly topical example of the benefit of teaching public health students computational skills is the widely referenced Johns Hopkins University resource for tracking the spread of the novel SARS COV-2 coronavirus (https://coronavirus.jhu.edu/map.html).

While the above focus on cross-discipline training is of paramount importance for future successful applications of AI and ML in the EHS, incentives for cross-disciplinary collaboration would have a more rapid impact and also would inform the integration of computer and data science into EHS curricula. Recognition of this opportunity by current EHS leadership and appropriate investments to achieve this goal would be of substantial benefit.

## What can AI and ML Do for Public Health and EHS?

### Challenge

A downstream consequence of the challenges detailed above is that the majority of researchers in the scientific community are still unaware of the benefits that AI and ML could provide when coupled with large, annotated, integrated datasets. A lack of familiarity with AI and ML as tools means that even when presented with examples of effective predictive models, potential end-users may not adopt them due to a lack of understanding, leading to decreased confidence in their utility. Further, without substantial investment in data curation and integration, the ability to apply AI and ML and build such models is severely limited.

### Recommendations

While these approaches are not yet mainstream, there are many examples of extremely successful implementations of combining big data with AI and ML to build high-performing predictive models, and such case studies should be widely distributed and serve as the catalysts for increasing support in these research areas. Beyond establishing the cyber-infrastructure to generate and store open, accessible data, select researchers, and government agencies are developing modeling approaches to effectively leverage those data. Aggregation of scholarly data into more structured computational models, such as quantitative structure activity relationship (QSAR)-based chemical predictions, demonstrates the efficacy of pipelines which turn decentralized data inputs into centralized models (Mansouri et al., 2016). Keeping these models in siloed communities is counter-productive, as the fundamental methods of model creation relies on open data provided by researchers. Drawing on standards for collaboration and sharing within the computer sciences, the National Institutes of Health (NIH) and the National Institute for Standards and Technology (NIST) have organized multiple hackathons and public-private partnerships to automate data extraction efforts and to create computational models that map the biological effects of chemical exposures (e.g., Kleinstreuer et al., 2018). Models resulting from such enterprises have surpassed the accuracy and efficiency of traditional, manual-labor driven animal testing (e.g., Browne et al., 2017). Projects such as the NCATS Biomedical Data Translator are designed to establish cross-cutting infrastructures to facilitate these data integration and modeling efforts (Austin et al., 2019).

If the above-mentioned hurdles can be overcome, big data, AI, and ML represent a huge opportunity for the expansion and application of effective environmental public health research, as discussed at a recent 2019 National Academies of Sciences workshop on “Leveraging Artificial Intelligence and Machine Learning to Advance Environmental Health Research and Decisions” (http://nas-sites.org/emergingscience/meetings/ai/). There exist a number of examples of researchers who are already bridging the fields of environmental public health, data science, and AI. The “AI for Earth” initiative partners Microsoft's data science acumen with researchers who have environmental expertise in the areas of agriculture, climate change, biodiversity, and water. Grants from AI for Earth have funded AI projects on population health model projections, image analysis for biodiversity, crop forecasting, climate-related landslide projections, modeling carbon sequestration, understanding global pathogen spread, and much more (Microsoft, 2018). AI and ML have facilitated a more nuanced and accurate understanding of climate patterns, improving the accuracy of forecasting extreme weather events to >90 percent (Cho, 2018). Researchers already use AI to improve air pollution forecasts (Fontes et al., 2014; Bellinger et al., 2017; Bai et al., 2018), disease diagnosis (Xiong et al., 2018), infectious disease monitoring (Milinovich et al., 2014 https://nextstrain.org/), tracking antibiotic resistance (Li et al., 2018), computational chemistry (Goh et al., 2017), exposure and chemical-mixture modeling (Bobb et al., 2014; Park et al., 2017; Vopham et al., 2018) and improving classification of climate regions (Liss et al., 2014). These innovative approaches and their successes are just the tip of the iceberg, and point to the potential benefit of sufficiently resourced investments in the application of AI and ML in environmental public health research.

## Conclusions and Recommendations

While grassroots innovation is increasing, top-down influencers within academia, government, industry, and funding bodies need to facilitate the conditions for AI to flourish in the fields of public and environmental health sciences. The philosophy of the “Fourth Industrial Revolution” (i.e., rapid technological advancement) is wildly different than the competition involved in typical academic research, as it requires enhanced interdisciplinary collaboration and universal data sharing and organization. As others have noted (Rubens et al., 2014), public health is generally slower than other scientific disciplines to embrace the use of advanced technologies. If we do not collectively aspire to change the framing of public health research and education, the discipline could impede its own progress. Technical talent will gravitate to the open, collective opportunities offered by private enterprise. To prevent this, change should percolate from those currently leading the field: researchers, educators, and practitioners need to understand the ingenious applications of emerging technologies and foster such opportunities within EHS research. Students should be encouraged, and even required, to explore these fields in core curriculums. During this time of unprecedented access to technical domains, cross-disciplinary training in computer science and EHS research will empower students and professionals alike to make meaningful contributions to perceivably the greatest revolution of their lifetime. We therefore propose actionable recommendations for leaders in the public and environmental health fields to implement and create an environment that will foster the data revolution (Table 1).

**Table 1:** Challenges and recommendations for fostering the big data revolution in environmental public health, summarized here and detailed in text.

**Challenge**	**Recommendation**
Data are not collected using controlled terminologies or standardized reporting formats.	Study data standards and ontologies should be designed and widely implemented. Such resources should be both specific to individual data types and coordinated across sources to optimize data utility and potential for integration.
Silos of information prevent access, integration, and effective data science analyses and applications.	Implement FAIR principles in data curation and development of scientific cyber-infrastructures. Establishing a culture of openness and data accessibility requires multiple nodes of cross-sector communication. Funding opportunities should emphasize this necessity.
Current environmental public health curricula do not emphasize data science skills.	Public health curricula should be data-minded and with ample resources for learning and improving technical skills. Establish tech-focused learning by offering relevant course, digital resources, and hosting speakers. Acquiring new skills needs to be celebrated and credentialed. Similarly, computer science programs should offer training in environmental health sciences and other biological application-oriented foci.
Lack of technical skills among environmental public health professionals leads to out-sourcing of data-science tasks and lack of adoption.	Training in AI, ML, and data science is not exclusive to universities or educational institutions. Continuing education courses, seminars, and conference sessions should provide environmental public health professionals with specialized resources and hands-on training in data science and new approach methodologies.
Public health culture does not prioritize data expertise and experimentation.	The scientific community must build an agile network of ambassadors and support curiosity and experimentation. Major institutions should appoint a team of experts within the organization to steer institutional culture and develop a wider network of expertise while also encouraging students, faculty, regulators, and professionals to apply data science tools in innovative ways.

## Author Contributions

SC, AA, and MV formulated the research question/premise, conducted research and literature reviews, and wrote the first draft of the manuscript. NK and RH contributed writing and research to sections of the manuscript. VV, NK, LJ, and RH assisted with conceptualizing the research question and contributed to manuscript editing. All authors contributed to manuscript revision and read and approved the final manuscript.

## Conflict of Interest

LJ is employed by Microsoft Corporation. The remaining authors declare that the research was conducted in the absence of any commercial or financial relationships that could be construed as a potential conflict of interest. The reviewer TL declared a past co-authorship with one of the authors NK to the handling editor.
